# A Deeper Look into the Biodiversity of the Extremely Acidic Copahue volcano-Río Agrio System in Neuquén, Argentina

**DOI:** 10.3390/microorganisms8010058

**Published:** 2019-12-29

**Authors:** Germán Lopez Bedogni, Francisco L. Massello, Alejandra Giaveno, Edgardo Rubén Donati, María Sofía Urbieta

**Affiliations:** 1 CINDEFI (CONICET-CCT LA PLATA, UNLP), Facultad de Ciencias Exactas, Universidad Nacional de La Plata, Calles 47 y 115, 1900 La Plata, Argentina; glopezbedogni@gmail.com (G.L.B.); massello.f@gmail.com (F.L.M.); donati@quimica.unlp.edu.ar (E.R.D.); 2 PROBIEN-CONICET-UNCo, Departamento de Química, Facultad de Ingeniería, Universidad Nacional del Comahue, Neuquén 8300, Argentina; agiaveno@hotmail.com

**Keywords:** acidic river, 16S rRNA biodiversity, extreme environment, acidophiles, Copahue-Caviahue

## Abstract

The Copahue volcano-Río Agrio system, on Patagonia Argentina, comprises the naturally acidic river Río Agrio, that runs from a few meters down the Copahue volcano crater to more than 40 km maintaining low pH waters, and the acidic lagoon that sporadically forms on the crater of the volcano, which is studied for the first time in this work. We used next-generation sequencing of the 16S rRNA gene of the entire prokaryotic community to study the biodiversity of this poorly explored extreme environment. The correlation of the operational taxonomic units (OTUs)s presence with physicochemical variables showed that the system contains three distinct environments: the crater lagoon, the Upper Río Agrio, and the Salto del Agrio waterfall, a point located approximately 12 km down the origin of the river, after it emerges from the Caviahue lake. The prokaryotic community of the Copahue Volcano-Río Agrio system is mainly formed by acidic bacteria and archaea, such as *Acidithiobacillus*, *Ferroplasma,* and *Leptospirillum*, which have been isolated from similar environments around the world. These results support the idea of a ubiquitous acidic biodiversity; however, this highly interesting extreme environment also has apparently autochthonous species such as *Sulfuriferula*, *Acidianus copahuensis,* and strains of *Acidibacillus* and *Alicyclobacillus*.

## 1. Introduction

Naturally acidic rivers are quite uncommon environments, their origins are majorly associated with volcanic activities. This singularity makes them attractive for many scientific areas. Río Tinto, in Huelva, Spain, is probably the most representative of these environments; a lot of works have focused on its biogeography, microbiology and it has even been used as an analogue of Mars [1]. The microbial community of Rio Tinto has been extensively studied having been found that its diversity is determined mainly by the high iron content along the river [2,3,4]. Among the dominant taxa reported are *Acidithiobacillus*, *Acidiphilium*, *Leptospirillum,* and, to a lesser extent, *Ferroplasma*, all of which are known as typical of acidic environments.

Contrary to naturally acidic rivers, acidic environments originated by anthropogenic activity, such as mining, have been deeply studied. The interest in these systems relies on the threat that acid mine drainage represents for freshwater resources and water scarcity in general. Reports in this topic are not only 16S/18S rRNA gene diversity assessments, but also characterizations and analyses of functions of interest such as iron and sulfur metabolism and heavy metal resistance mechanisms. [5,6,7,8,9].

Rio Agrio, in Neuquen, Argentina, is an acidic river affected by the still active Copahue volcano. Copahue volcano is an active stratovolcano whose latest eruption was recorded in 2018. Previous biodiversity assessment on the Rio Agrio [10] revealed the presence of some bacteria and archaea, which could not be completely classified, and which seemed to be indigenous of this system. However, the mentioned taxa known as typical of acidic environments were also found, raising a question about the ubiquity and dispersion of these acidophilic taxa.

Biogeography is the study of the spatial distribution of living species. In the case of microorganisms, the importance of geographical distribution has been generally despised, mainly because it has been considered that, due to their ease of dispersion, their ability to pass into dormant stages and their rapid growth rates, microorganisms are able to overcome any geographical barrier and distance. Therefore, the microbial distribution turns out to be a result of the environment selection. However, since next-generation sequencing techniques became more accessible, many studies have questioned this hypothesis and propose the existence of spatial patterns of distribution; moreover, different authors showed that the genetic distance between groups of microorganisms can be correlated with the geographic distance between them [11,12]. Regarding acidic environments, there are very few specific works focusing on geographical distribution, and those that deal with the phylogeny of the communities support the hypothesis that species selection is mainly driven by environmental factors, as species highly related have been found in very similar but distant environments [13,14]. Even though our work does not intend to deepen the biogeography of acidic prokaryotes, the study of the Copahue volcano-Río Agrio system could serve as another model to assess the microbial diversity, the correspondence with environmental parameters, and the concurrence of representative taxa in similar environments.

This work represents the first assessment of the prokaryotic community of the pristine and naturally acidic system of Copahue volcano-Rio Agrio (Neuquen, Argentina) using high-throughput sequencing. It deepens the knowledge of this little-explored environment, opens the door for future functional characterizations, and promotes the identification of novel indigenous species.

## 2. Materials and Methods

### 2.1. Site Description, Sampling, and Phisicochemical Determinations

**Figure 1:**
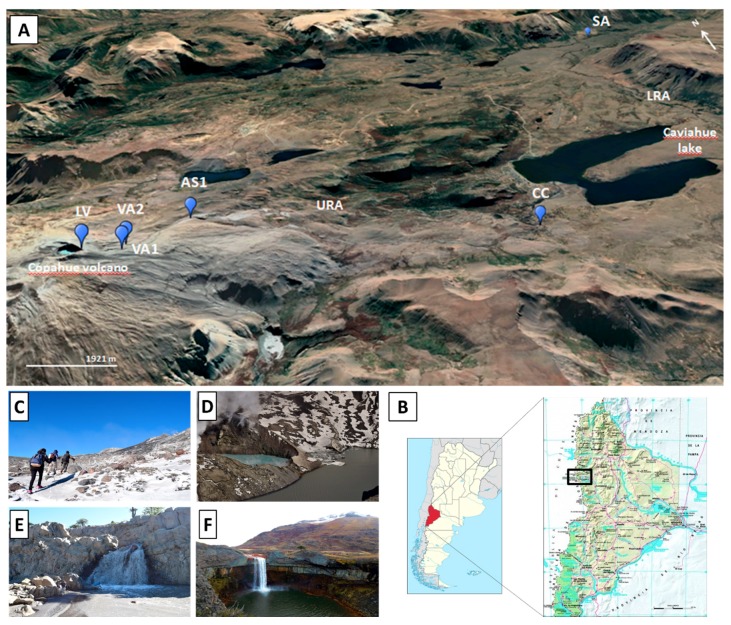
**(A**) Satellite image of the sampling area, the sampling points are marked in blue. (**B**) Location of the Copahue volcano-Río Agrio system in Argentina and in Neuquén province. (**C**–**F)**: Images of the sampling points.

Figure 1 shows the location of the sampling points along the Rio Agrio course in Neuquen, Argentina (Figure 1A,B). Rio Agrio originates as a very narrow water course flowing through the rocks approximately 100 m below the crater of the Copahue volcano (2965 m a.s.l.) at two acidic hot springs named “Vertiente Agrio 1” and “Vertiente Agrio 2” (VA1 and VA2). A few meters downstream, these two sources meet and form the Upper Rio Agrio (URA). The URA flows downhill Copahue volcano for 13.5 km where it discharges in the Caviahue lake at 1600 m a.s.l. In its path, it receives the input of different snowmelt origin rivers and streams and it forms various waterfalls; in this study, this part of the URA is represented by sampling points AS1 and “Cascada de la Culebra” (CC) (Figure 1E). The pH of the water at the origin of the river is highly acidic and remains stable throughout the course of the URA, ranging from 0.3 to 2.3. On the other hand, the temperature in the origin varies greatly, depending on the volcanic activity, with registered values between 70 °C and 20 °C, and it gets colder as soon as the river leaves its geothermal origin, mainly due to the input of tributary courses and melting snow. After emerging from the Caviahue lake, the river, now called Lower Rio Agrio (LRA), is wider, has a larger flow, and is less acidic. Such changes, especially the pH increase, produce abundant precipitation of ferric minerals, which gives the red-orange color characteristic of the place [15]. This portion of Río Agrio is represented by a sample collected at a great waterfall called Salto del Agrio (SA) (Figure 1F).

The Copahue volcano harbors another unusual extreme environment: The acidic lagoon that forms in its crater (sample LV) (Figure 1D). Interestingly, it is not always there; its presence depends on the rain regime, the snow melt, and the volcanic activity. Even though it is not geologically associated with the river, it is a rare extreme environment that is part of the Copahue geothermal system. The lagoon presents higher mineralization than the river due to the volcanic gases, it is markedly acidic, with pH values registered between 0.2 and 1.1, while temperature fluctuates between 10 °C and water-boiling temperature when measured in eruptive periods [16]. The level of the lagoon modifies constantly, and it has even disappeared after the eruption in 2000. Thereafter, it increased its level; however, until March 2018, it had not been possible to collect any samples from there.

For the river samples, 3 L of water were collected in sterile plastic bottles while in the lagoon, it was possible to collect only one litter. Temperature, pH, and redox potential were measured in situ with a Hanna HI 8424 NEW portable instrument (Hanna Instruments, Woosoncket, RI, USA) properly calibrated against calibration standards.

Samples were filtered using 0.22 µm pore sizes cellulose membranes when arrived at the laboratory and washed with pH 2 sterile water and phosphate buffered saline solution (PBS) to remove any acidic water containing heavy metals that may cause DNA hydrolysis [17]. Membranes were stored at −20 °C until DNA extraction and the filtrates were used for further physicochemical determinations done at Laboratorio de Geoquímica at CIG (Centro de Investigaciones Geológicas, CONICET-UNLP, La Plata, Buenos Aires, Argentina). Ca, Na, and K were measured by atomic absorption spectrophotometry; Sb, As, Be, Cd, Co, Cr, Sn, Fe, Mn, Ni, Pb, Se, Zn, Li, Rb, Sr, and V were measured by ICP-MS. Nitrates and sulfates were determined by UV-visible spectrophotometry, while chlorides were determined by titration and conductivity was measured using an specific electrode. Organic matter was determined by the chemical oxygen demand by oxidation with KMnO_4_ (Ma, 2016) [18].

### 2.2. DNA Extraction and Sequence Processing

DNA extraction was performed using the membranes following the cetyl trimethylammonium bromide (CTAB) (G-Biosciences, St Louis, MO, USA) protocol. The DNA quality was estimated using Nanodrop (Thermo Scientific, Wilmington, DE, USA). Genomic DNA extracted was send to MR. DNA (Molecular Research Laboratory, TX USA) where the V3-V4 hypervariable regions of prokaryotic 16S rRNA genes were amplified using the primer set 341F (CCTACGGGNBGCASCAG) and 806R (GGACTACNVGGGTWTCTAAT) and sequenced with an Illumina MiSeqplatform (Illumina, San Diego, CA, USA).

The raw sequence data was processed using the MOTHUR package [19] following the standard operating procedure [20]. The sequences obtained were quality trimmed according to the following parameters: Quality score higher than 25, sequence length of 429 bp, no ambiguous bases, and 8 maximum homopolymers. VSEARCH algorithm was used to detected chimeras and remove them appropriately. Good-quality sequences were preclustered allowing for up to 2 differences between sequences. OTUs were composed at 97% of nucleotide identity. Singletons were removed and taxonomic assignments were archived at the 80% threshold using the Silva non-redundant v132 database [21]. After processing, 171,093 sequences corresponding to 1114 OTUs were obtained. The data have been deposited at BioProject accession number PRJNA542136 in the NCBI BioProject database (https://www.ncbi.nlm.nih.gov/bioproject/).

The most abundant OTUs were compared against the NCBI nucleotide non-redundant database and the 16S ribosomal RNA sequence database using the BLAST tool.

### 2.3. Statistical Analyses

Statistical analyses were performed using R packages [22]. Alpha diversity indices (Shannon and Simpson) were calculated with the BiodiversityR package [23]. CCA and rarefaction curves were calculated using the PAST software [24].

### 2.4. OTUs Global Ocurrence

The representative sequences of the first 20 OTUs were blasted against the nucleotide database of NCBI. We used the megablast program and the database was filtered for sequences of length between 1000 and 2000 nucleotides. The best matches of each OTU were selected as follows: Only alignments that covered the entire query sequence were kept; the percentage of identity threshold was set allowing only one mismatch or gap. GenBank data of the selected sequences were downloaded and their isolation information was extracted. Sequences that had not enough metadata were discarded. The environments where the sequences were retrieved were classified in eight categories: Acid mine drainage (AMD), mine, wastewater, riverine, volcanic, hydrothermal, bioprocess, and other natural environment. Matched sequences were approximately located in a map using leaflet R package [25]. Sequences whose locations were not accurately specified were located in the capital of the corresponding country.

A chord diagram representing the environment in which each OTU sequence has been found was constructed using the circlize R package [26].

## 3. Results

### 3.1. Physicochemical Description of Rio Agrio

Rio Agrio is one of the few naturally acidic rivers, Table 1 shows the main physicochemical characteristics of the sites analyzed in this study. The two geothermal sources of the river, VA1 and VA2, present pH values around 2, however they differ in temperature (VA1 41.0 °C and VA2 28.8 °C), something already observed in previous samplings [27]. The variation in temperature is probably related to the fact that VA2 is more exposed to the cold wind and the melting snow. The next point, AS1, is located a few kilometers downhill, where the URA receives the input of different tributary courses mainly from snowmelt that dilute the original geothermal composition of the waters and set the temperature in the values that it has in its entire course, between 12 °C and 16 °C. Such dilution does not affect pH, which remains around 2, but it generates a slight decrease in the concentration of soluble anions and cations as well as conductivity (Table 1). Following the course of the river, the sampling point CC has a pH of 2.5, while ions concentrations and conductivity decrease to about half its value. The last point sampled, SA in the LRA, presents less acidic waters (pH 3.8) and ion concentrations and conductivity are more of an order of magnitude lower than at the origin; on the other hand, temperature remains around 12 °C.

Regarding metal cations in Río Agrio, Na^+^, K^+^ and Ca^2+^ showed concentrations over the average reported for freshwater (1–150 mg/L, 1–10 mg/L, and 10–250 mg/L, respectively [28]) except in SA. Iron was the only other metal with significantly high concentrations; it started with almost 1000 mg/L in VA1 and decreased to near 10 mg/L in SA, due to dilution and pH increase, which causes its precipitation. The most relevant anions were chlorine and sulfate, both more concentrated than in freshwater (10–250 mg/L and 2–150 mg/L, respectively). In the case of sulfate, the concentration close to the origin of the river (VA1, VA2, and AS1) was near the average value in sea water (3000 mg/L) [28].

The physicochemical conditions of the water of the crater lagoon were quite different; it was less acidic than the origin of the river, with a pH value of 2.8, probably due to the constant presence of snow, much colder (4 °C), and all anions and cations were in much lower concentrations (Table 1).

### 3.2. Sequencing and Diversity Analysis

#### 3.2.1. Biodiversity Indexes

The 171,093 16S rRNA gene fragments processed were used to calculate the rarefaction curves and the diversity indexes of the six sampling points along the Copahue volcano-Río Agrio system. All the rarefaction curves reached a plateau (Figure S1), therefore total species richness was well represented in all the samples. Alpha diversity analysis was performed calculating the Shannon–Wiener’s and Simpson’s indexes (Table 2). The Shannon–Wiener function depends on the number of species (richness) and their abundance; it gives more weight to richness than evenness being a better estimator than a simple count of species (observable). The Simpson index, on the other hand, is a dominance index; it is more sensitive to evenness and less sensitive to richness than Shannon. The Simpson index decreases as richness increases, so it is frequently reported as the inverse-Simpson (1/Simp).

The highest diversity of OTUs was found in SA according to the value of the Shannon–Wiener index and to the number of observed OTUs. SA was also the most even, with a much higher value of the 1/Simp index than the other points of the river. On the other hand, the less diverse and even of the samples was LV. Finally, the points of the URA (VA1, VA2, AS1, and CC) did not show significant differences of diversity and evenness according to the calculated indexes (Figure 3).

#### 3.2.2. Taxonomic Composition of the Prokaryotic Diversity

**Figure 2:**
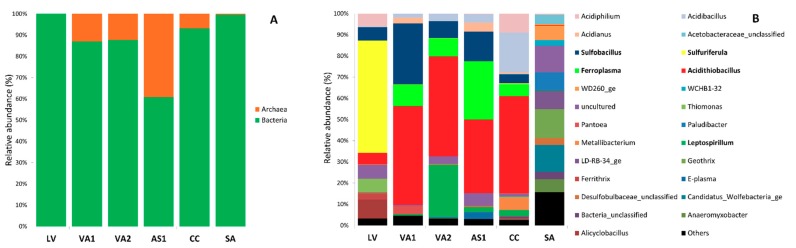
Relative abundances of the 16S rRNA sequences classified according Silva database. (**A**) Domain; (**B**) genera with relative abundances over 2%. Genera with abundances below 2% were grouped as “Others”.

The metagenomic dataset of the six points after processing consisted of 171,093 16S rRNA gene fragments; of this total, 148,890 sequences (87.02%) correspond to the domain Bacteria and 22,203 (12.98%) belong to the domain Archaea (Figure 2A). At the Phylum level, we found that 24 phyla describe the diversity of Bacteria while the Archaea diversity was only represented by four phyla. Figure 2B shows the relative distribution of the genera with abundances over 2%. Supplementary Table 1 presents a description of the OTUs up to 1% of relative abundance in the six points sampled, including percentage of abundance, phylogeny at genus level (confidence percentage of the taxonomic classification between brackets), closest BLAST hits, their accession number, percentage of similitude, and relevant characteristics of the environments where they were obtained if possible.

#### 3.2.3. Bacterial Diversity

Proteobacteria was the most abundant phylum with more than 50% of the sequences in all the points along the Copahue volcano-Río Agrio system. Within this group, *Acidithiobacillus* was the most represented genus in samples VA1, VA2, AS1, and CC, with relative abundances of 45.22%, 46.37%, 34.14%, and 43.20%, respectively; in LV, the genus *Sulfuriferula* had over 51% of the total abundance while in SA, the distribution was more equitable with the genera WD260 (5.68%) and *Anaeromyxobacter* (5.06%) as the most represented among Proteobacteria. The phylum Firmicutes was also present in all the course of the river; however, its abundance changes at each point. In LV, VA1, and CC, it was the second most abundant phylum, represented by the genera *Alicyclobacillus* (8.71% in LV), *Sulfobacillus* (28.02% in VA1), and *Acidibacillus* (17.85% in CC). In VA2 and AS1, Firmicutes were less abundant; in both points, *Sulfobacillus* was the most represented genus with abundances of 8.21% in VA2 and 13.87% in AS1. In SA, Firmicutes represented less than 3% of the total bacterial community. Among the studied sites, only in VA2, AS1, and CC the phylum Nitrospirae was present, represented in all cases by the genus *Leptospirillum*; particularly, in VA2, it was the second dominant genus with 24.34% of abundance, while it accounted for less than 3% in the other two sites. In the case of SA, the diversity was completed by the phyla Bacteroidetes (23.35%), represented by the genera LD-RB-34 (7.50%) and *Paludibacter* (6.82%); Acidobacteria (12.71%), represented by the genus *Geothrix* (10.92%); and the phylum Patescibacteria (12.62%) with the genus Candidatus *Wolfebacteria* (10.35%).

#### 3.2.4. Archaeal Diversity

The archaea of Río Agrio belonged to four phyla: Euryanchaeota, Crenarchaeota, Nanoarchaeota, and Thaumarchaeota. The last one was only represented by a unique OTU and its relative abundance was practically zero. Nanoarchaeota were present in a very low proportion (0.6% of the total archaeal reads) in CC and SA, and in both cases, were only represented by the genus *Woeserachaeia*. The genus *Acidianus* covered the total reads of the phylum Crenarchaeota (which represented the 10.3 % of the archaea) and was founded all along URA (samples VA1, VA2, AS1, and CC). Euryarchaeota was the main archaeal phyla in all Río Agrio (89.1% of abundance), being present in all the sampled sites, mostly in VA1, VA2, AS1, and CC, by the genera *Ferroplasma,* and the less abundant group “E-plasma”.

### 3.3. Statistical Analysis of the Physicochemical Parameters and the Prokaryotic Biodiversity of the Copahue Volcano-Río Agrio System

Canonical correspondence analysis (CCA) is a correspondence analysis of a site/species matrix where each site has given values for one or more environmental variables (temperature, pH, etc.). The ordination axes are linear combinations of the environmental variables. CCA is thus an example of direct gradient analysis, where the gradient in environmental variables is known a priori and the species abundances are a response to this gradient [29].

**Figure 3:**
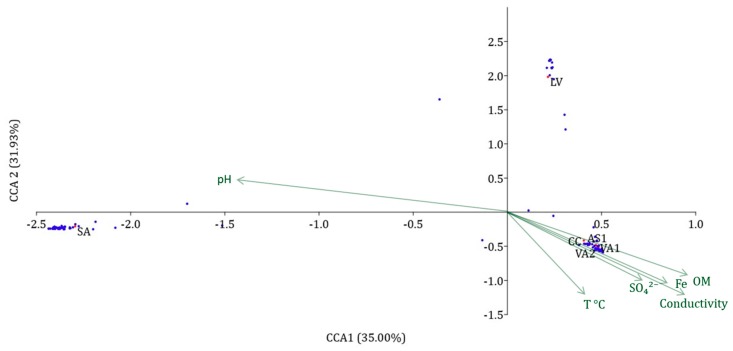
Canonical correspondence analysis (CCA) of the samples of the Copahue volcano-Rio Agrio system and their physicochemical variables. The variability explained by each component is shown in the axis labels. Red points and black letters represent the sampled sites. Light green arrows indicate the physicochemical variables. Blue dots represent the OTUs with more than 1% abundance.

Figure 3 shows the distribution of the physicochemical variables of the first two components of the CCA, which explained the 66.93% of the total variance observed for the data of the Copahue volcano-Río Agrio system. Both axes explained the information in similar proportion and clearly separated the sites in three different habitats regarding the physicochemical characteristics. One group is formed by all the points collected in the URA (VA1, VA2, AS1, and CC), which positively correlates with temperature, organic matter, conductivity, iron, and sulfate concentrations and negatively correlate with pH. It is worth noticing how the four sampling points of the URA as well as the symbols representing the OTUs are very close together meaning that they are very similar in both environmental characteristics and biodiversity. On the other hand, the crater lagoon is clearly separated from the points of the URA, especially by its negative correlation with temperature, organic matter, conductivity, iron, and sulfate concentrations; it also shows its own biodiversity with only few OTUs shared with the other groups. The last group only contain the point SA, which is the only one that correlates positively with pH and negatively with the other variables and, like the other groups, it has OTUs very close together, sharing almost no taxa with LV or URA.

## 4. Discussion

### 4.1. Influence of Geochemistry of the Río Agrio in Its Prokaryotic Biodiversity

Río Agrio originates as a natural acidic river due to the volcanic gases (H_2_SO_4_, HF, HCl) discharged by the hot springs that give it origin [30]. In this study, the pH measured at the origin (samples VA1 and VA2) was two (our group had measured pH values close to 1 in other campaigns [10]) and it remains almost constant all through the course of URA, in spite of the dilution effect produced by receiving multiple neutral tributary courses mainly of snowmelt origin. The dilution of URA after the origin is reflected in a decrease in conductivity, anions, and cations concentrations that starts in AS1 and becomes more drastic in CC. In other natural acidic rivers, for example, Río Tinto in Spain, the waters are acidic in all its course mainly because of the heavy load of Fe (III) (up to 20 g/L), which forms a buffer system [31]. In Río Agrio, total iron concentration is less than 1 g/L in the origin and diminishes to 0.01 g/L in LRA, making it impossible to exercise the same buffer capacity. The most possible explanation for the acid condition is the metabolic activity of different microbial species, such as *Acidithiobacillus*, *Acidiphilium*, *Sulfobacillus,* and *Acidibacillus*, capable of oxidizing sulfur and sulfur compounds highly abundant in the area due to the geochemistry and the volcanic activity of the area.

### 4.2. Characteristics of the Prokaryotic Community of the Copahue Volcano-Río Agrio System

Our research group has been working on the assessment of the biodiversity of Río Agrio for the last 20 years. At the beginning, we relied only on culture depending techniques which allowed the identification of several acidophilic iron and/or sulfur oxidizing species [32,33]. These results encourage us to perform more comprehensive assessments of prokaryotic biodiversity, such as cloning and sequencing of 16S rRNA genes of bacteria and archaea [10]. Despite finding a quite rich extreme biodiversity, both approaches were highly incomplete in the light of next-generation sequencing techniques. Thus, the main goal of this work was to complete our previous efforts by doing a deeper assessment of the prokaryotic biodiversity of Río Agrio, and for the first time of the acidic lagoon at the crater of Copahue volcano, using high-throughput sequencing.

The results of this new approach, supported by the CCA analysis (Figure 3), showed that the Copahue volcano-Río Agrio system clearly has three environments inhabited by different microbial communities: The crater lagoon, the URA, and Salto del Agrio. The crater lagoon (LV) was dominated by *Sulfuriferula,* which are neutrophilic and mesophilic species able to oxidize a variety of sulfur compounds [34]. The remaining species, which present lower abundances, were mostly acidophilic species, mesophiles, or moderately thermophiles related to sulfur or iron metabolism and found in other acidic environments including our previous study of Río Agrio [10] (see Table S1 in Supplementary Material). Among them, *Ferrithrix*, an extremely acidophilic, moderate termophilic and obligate heterotrophic iron oxidizing genus [35], and *Thiomonas*, a moderate acidophilic, mesophilic, quimioheterotrophic sulfur oxidizing genus, were only detected at this sampling station. No archaea were found at the crater lagoon. The occurrence of species in pH and temperature conditions apparently distant from their growth optimum may be explained considering the changing nature of this point of the system. It could also be expected that the relative abundances vary greatly with pH and temperature modifications favoring the better adapted species. In the particular case of *Sulfuriferula,* it has also been detected by high-throughput sequencing in an acidic water reservoir (pH 2.85, temperature 13.2 °C) of an open-cast mine for polymetallic ores in Eastern Siberia [36]. The genera *Ferrithrix* has been reported in the volcanic island of Marion located, in the South African zone of sub-Antarctica where the annual temperature mean ranges 5–6 °C [37], and even in the rock coatings in the glacial valley of the Northern Caledonide Mountains of Swedish Lapland where the annual temperature mean is −1 °C [38]. Finally, it is worth noting that the sequences obtained from LV present identity percentages lower than 97% with cultured characterized species, thus they could be potential autochthonous novel species with different optimal growth conditions. Our efforts are now concentrated in their isolation and characterization.

On the other hand, the URA, constitutes another environment with a relatively stable microbial community. This community was dominated by *Acidithiobacillus* species (between 37% and 48% of abundances in the four sampling stations, see Figure 2), *Sulfobacillus*, *Ferroplasma*, *Leptospirillum,* and *Acidibacillus*, which are acidophilic, mesophilic, or moderately thermophilic, sulfur and/or iron oxidizing species. Among these genera, some of the OTUs found have the peculiarity of being distantly related to cultivated members (97% or less) but being 99–100% similar to different uncultured sequences found in acidic environments such as Rio Tinto, hot springs, and mine tailings (Table S1). Particularly, all the OTUs affiliated with the genus *Acidibacillus* were 99% similar to uncultured clones retrieved from Río Agrio but distantly related to cultivated species. In the same way, the archaeal OTUs affiliated to the family Thermoplasmataceae were 99–100% similar to sequences retrieved from the Copahue-Caviahue system and from an acid mine drainage of a metal-rich abandoned tailing ponds in Tongling, China [39]; however, they were around 90% similar to cultivated species of the genus *Thermoplasma*. In both cases, it may indicate the presence of potential indigenous novel species or even a new characteristic genus of acidic environments.

*Acidianus copahuensis*, the thermoacidophilic crenarchaeota isolated from the Copahue geothermal system [40], was found only in VA1 and AS1 with 2.6% and 4.2% abundances, respectively, and the genus *Acidiphilium*, represented by acidophilic, mesophilic, chemoorganotrophic, and sulfur oxidizing species [41], only appeared in LV and CC (5.8% and 7.9% of abundances, respectively). In CC, there were also found two OTUs (5.4% of abundance) with 98–100% of similarity to *Metallibacterium scheffleri*, which is a mesophilic rod-shaped acidophile able to grow only on casein as a carbon source. It has been isolated from an acidic biofilm in a pyrite mine [42] and similar sequences have been retrieved from different acidic environments including Rio Tinto and Rio Agrio [3,10]. Even though *M. scheffleri* is a moderate acidophile with optimum growth pH around 5.5, it is able to survive in more acidic conditions because, as a product of the metabolism of casein, it releases ammonium, which elevates the pH of its vicinity. The question that still needs to be answered is how a species with such strict nutritional requirements can survive in environments with very low organic matter like Río Agrio, Río Tinto, or mining zones.

Finally, Salto del Agrio had a completely different and much more diverse microbial community with no clear dominant members. There was a higher abundance of anaerobic species and no significant occurrence of the acidophilic species found in the URA or the crater lagoon. Such differences could be explained considering the higher pH, lower Eh, and the iron concentration at this site. Despite the low abundance of the most common acidophiles in this community, species related to *Acidithiobacillus*, *Acidiphilium,* and *Leptospirillum* have been cultured from samples collected from Salto del Agrio [33,43].

### 4.3. Global Occurrence of the OTUs

**Figure 4:**
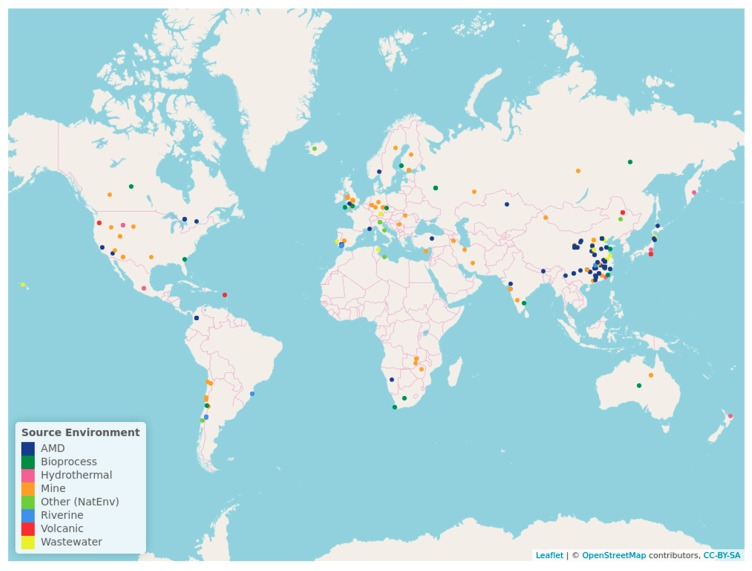
OTUs global occurrence. The colors of the points indicate the source of the sequences related to the first 20 OTUs of the Copahue volcano-Río Agrio system.

**Figure 5:**
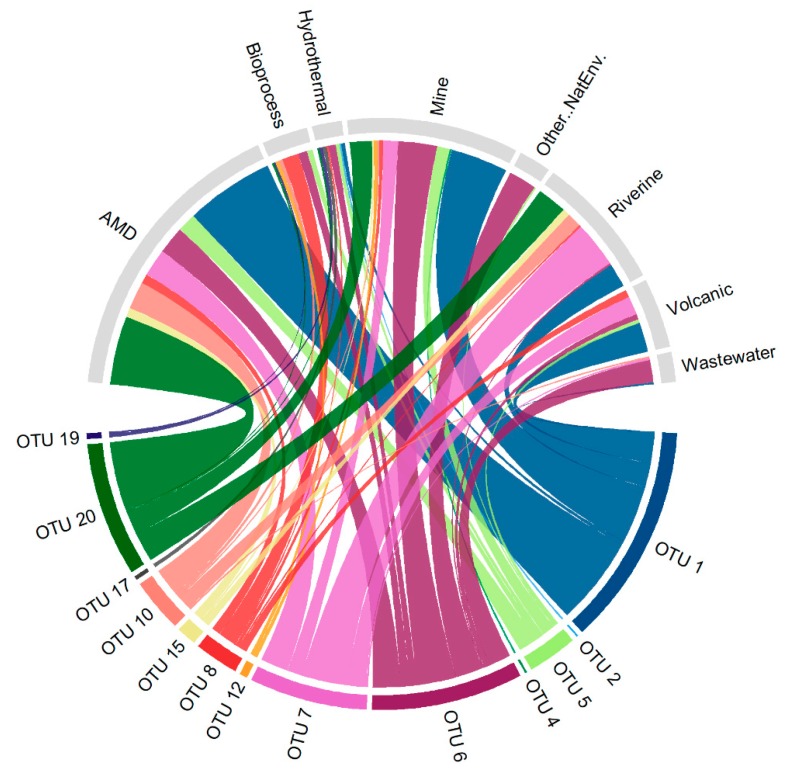
Chord diagram showing the occurrence of the OTUs in the source environment defined in the text and in Figure 4.

When analyzing the sequences related to the OTUs found in the Copahue volcano-Río Agrio system, we evidence that most of them were retrieved or isolated from similar acidic environments, apart from our previous studies of Copahue Geothermal system [10,40,44] (Table S1). For instance, the OTUs associated with *Acidithiobacillus*, *Leptospirillum*, Clostridiales, the Deltaproteobacteria genus Sva0485_ge, and the archaeal OTUs associated with *Ferroplasma*, E-plasma, and Thermoplasmataceae were 100–99% similar to sequences retrieved from an acid mine drainage from metal-rich abandoned tailing ponds in Tongling, China [39]. Similarly, sequences related to the OTUs affiliated with *Acidithiobacillus*, *Leptospirillum*, *Acidibacillus*, *Metallibacterium*, Deltaproteobacteria genus Sva0485_ge, *Acidiphilium*, Thermoplasmataceae, and E-plasma were obtained from the naturally acidic river Rio Tinto in Spain [3,4], while other sequences associated with *Leptospirillum*, *Acidiphilium*, *Metallibacterium*, Thermoplasmataceae, and E-plasma were retrieved from the extremely acidic environment of the Parys Mountain copper mine in United Kingdom [45]. To locate the sites where the microorganisms associated to the Copahue volcano-Rio Agrio OTUs have been isolated, we constructed a map (Figure 4) and classified those environments in eight categories: Acid mine drainage (AMD), mine, wastewater, riverine, volcanic, hydrothermal, bioprocess, and other natural environment. This map clearly shows that the points are mostly concentrated in certain geographical locations. We also made a chord diagram (Figure 5) that represents the occurrence of each of the 20 top OTUs in the eight type of environments. The figure shows that most of the representative sequences have been detected in acidic environments, as AMD, mine, and riverine (which mostly includes Río Tinto and Río Agrio). It also confirms that certain species, such as *Acidithiobacillus* (OTUs 1, 5, 6, and 10), *Leptospirillum* (OTU 7), and *Acidiphilium* (OTU 20) have strong presence in diverse acidic environments. The OTUs that do not appear in the chord diagram have no related sequences in the NCBI data base (considering the restrictions explained in the Material and Methods Section 2.4) and in certain cases could be considered autochthonous of the Copahue volcano-Rio Agrio system. The only two archaeal OTUs, OTU 2 (*Ferroplasma*) and OTU 17 (*Acidianus*), have only one chord, meaning that within the parameters selected, they have only one representative sequence, and in both cases it was retrieved from the Copahue geothermal system (see Table S1); therefore, they could also be considered autochthonous of this system.

Other studies have shown the presence of certain species in diverse acidic environments; for instance, Holanda and co-workers [13] isolated and characterised eight strains of the acidophilic, mineral oxidising bacteria *Acidibacillus* spp. from mine sites and geothermal environments in very different global locations. However, as mentioned above, there are certain OTUs found in this study that have very low similarity matches in the available databases and seem to be autochthonous of the Copahue geothermal area. For instance, OTU 3 (*Sulfuriferula*), OTUs 11 and 14 (*Acidibacillus*), OTU 13 (*Alicyclobacillus*) (Table S1), and the confirmed case of OTU 17, are associated with the thermoacidophilic archaeon *Acidianus copahuensis* that has been isolated from the Copahue geothermal system [40] and, to date, it has not been reported anywhere else in the world.

## 5. Conclusions

This work is the first study that uses next-generation sequencing to assess the prokaryotic diversity of Copahue volcano-Río Agrio system and, also, is the first to reveal the biodiversity in the extreme environment of the Copahue volcano crater lagoon. Río Agrio harbors many acidophilic species of bacteria and archaea with strong presence in other similar acidic environments; however, it also possesses apparently autochthonous species such as *Sulfuriferula*, *Acidianus copahuensis,* and strains of *Acidibacillus* and *Alicyclobacillus*. Our efforts now are directed to the isolation and characterization of these possible new species.

## Figures and Tables

**Table 1:** Physicochemical data and geographic location of the six samples collected in the Copahue volcano-Rio Agrio system.

Site	LV	VA1	VA2	AS1	CC	SA
Coordinates	S 37° 51′ 21′’	S 37° 51′ 23′’	S 37° 51′ 18′’	S 37° 50′ 59′’	S 37° 53′ 08′’	S 37° 48′35′’
	W 71° 09′ 29′’	W 71° 09′ 04′’	W 71° 08′ 59′’	W 71° 08′ 00′’	W 71° 04′ 02′’	W 70° 55′33′’
pH	2.79	1.92	2.00	2.06	2.50	3.89
T (°C)	4.00	41.00	28.80	12.00	15.90	12.60
Conductivity (µs/cm)	1402.00	27,100.00	20,600.00	16,990.00	7770.00	350.00
Eh (mV)	ND	365.00	398.00	382.00	405.00	310.00
Cl^−^ (mg/L)	0.70	5562.10	3771.73	3103.32	978.74	30.79
NO₃^−^ (mg/L)	11.20	8.82	49.28	49.45	23.34	0.36
SO₄²¯ (mg/L)	78.49	2841.01	2616.18	2479.92	98.79	20.64
Organic matter (mg/L) *	4.23	8.84	12.78	23.53	13.22	2.91
Ca (mg/L)	24.38	565.82	547.06	418.90	232.58	13.44
Na (mg/L)	22.50	1050.00	620.00	480.00	257.00	14.20
K (mg/L)	2.70	60.00	41.00	58.00	25.00	3.60
Fe (mg/L)	55.48	998.41	780.08	693.06	241.61	12.92
Mn (mg/L)	1.08	45.71	29.55	24.83	11.79	0.48

* organic matter is expressed in mg/L as Chemical Oxygen Demand. ND: not determined.

**Table 2:** Alpha diversity analysis.

	LV	VA1	VA2	AS1	CC	SA
Observed OTUs	290	311	280	331	504	681
Shannon-Wiener	2.276	2.445	2.483	2.505	2.925	4.326
Inverted Simpson	3.832	6.350	5.683	6.162	5.787	28.498

## Supplementary Materials

The following are available online at https://www.mdpi.com/2076-2607/8/1/58/s1. Table S1: Description of the OTUs up to 1% of relative abundance in the six points sampled, including percentage of abundance, phylogeny at genus level (confidence percentage of the taxonomic classification between brackets), closest BLAST hits, their accession number, percentage of similitude and relevant characteristics of the environments where they were obtained if possible. Figure S1: Rarefaction curves.

## References

[1] Amils R., González-Toril E., Fernández Remolar D., Gómez F., Aguilera A., Rodríguez N., Malki M., García-Moyano A., Fairén A., de la Fuente V. (2007). Extreme environments as Mars terrestrial analogs: The Rio Tinto case. Planet. Space Sci..

[2] González-Toril E., Llobet-Brossa E., Casamayor E.O., Amann R., Amils R. (2003). Microbial ecology of an extreme acidic environment, the Tinto River. Appl. Environ. Microbiol..

[3] Garcia-Moyano A., Gonzalez-Toril E., Aguilera A., Amils R. (2012). Comparative microbial ecology study of the sediments and the water column of the Rio Tinto, an extreme acidic environment. FEMS Microbiol. Ecol..

[4] Sánchez-Andrea I., Rodríguez N., Amils R., Sanz J.L. (2011). Microbial diversity in anaerobic sediments at Rio Tinto, a naturally acidic environment with a high heavy metal content. Appl. Environ. Microbiol..

[5] Bomberg M., Mäkinen J., Salo M., Kinnunen P. (2019). High Diversity in Iron Cycling Microbial Communities in Acidic, Iron-Rich Water of the Pyhäsalmi Mine, Finland. Geofluids.

[6] Aguinaga O.E., Wakelin J.F., White K.N., Dean A.P., Pittman J.K. (2019). The association of microbial activity with Fe, S and trace element distribution in sediment cores within a natural wetland polluted by acid mine drainage. Chemosphere.

[7] Gao P., Sun X., Xiao E., Xu Z., Li B., Sun W. (2019). Characterization of iron-metabolizing communities in soils contaminated by acid mine drainage from an abandoned coal mine in Southwest China. Environ. Sci. Pollut. Res..

[8] Zhang X., Tang S., Wang M., Sun W., Xie Y., Peng H., Zhong A., Liu H., Zhang X., Yu H. (2019). Acid mine drainage affects the diversity and metal resistance gene profile of sediment bacterial community along a river. Chemosphere.

[9] Leitholf A.M., Fretz C.E., Mahanke R., Santangelo Z., Senko J.M. (2019). An integratemicrobiological and electrochemical approach to determine distributions of Fe metabolism in acid mine drainage-induced “iron mound” sediments. PLoS ONE.

[10] Urbieta M.S., González-Toril E., Aguilera A., Giaveno M.A., Donati E. (2012). First prokaryotic biodiversity assessment using molecular techniques of an acidic river in Neuquén, Argentina. Microb. Ecol..

[11] Jones D.S., Schaperdoth I., Macalady J.L. (2016). Biogeography of sulfur-oxidizing Acidithiobacillus populations in extremely acidic cave biofilms. ISME J..

[12] Fontaneto D., Hortal J., Ogilvie L.A., Hirsch P.R. (2012). Microbial biogeography: Is everything small everywhere. Microbial Ecological Theory: Current Perspectives.

[13] Holanda R., Hedrich S., Nancucheo I., Oliveira G., Grail B.M., Johnson D.B. (2016). Isolation and characterisation of mineral-oxidising ‘Acidibacillus’ spp. from mine sites and geothermal environments in different global locations. Res. Microbiol..

[14] Menzel P., Gudbergsdóttir S.R., Rike A.G., Lin L., Zhang Q., Contursi P., Moracci M., Kristjansson J.K., Bolduc B., Gavrilov S. (2015). Comparative metagenomics of eight geographically remote terrestrial hot springs. Microb. Ecol..

[15] Urbieta M.S., Willis-Porati G., Segretín A., González-Toril E., Giaveno M., Donati E. (2015). Copahue geothermal system: A volcanic environment with rich extreme prokaryotic biodiversity. Microorganisms.

[16] Varekamp J.C., Ouimette A., Herman S., Flynn K.S., Bermúdez A.H., Delpino D.H. (2009). Naturally acid waters from Copahue volcano, Argentina. Appl. Geochem..

[17] Herrera A., Cockell C.S. (2007). Exploring microbial diversity in volcanic environments: A review of methods in DNA extraction. J. Microb. Methods.

[18] Ma Y.J., Tie Z.Z., Zhou M., Wang N., Cao X.J., Xie Y. (2016). Accurate determination of low-level chemical oxygen demand using a multistep chemical oxidation digestion process for treating drinking water samples. Anal. Methods.

[19] Schloss P.D., Westcott S.L., Ryabin T., Hall J.R., Hartmann M., Hollister E.B., Sahl J.W. (2009). Introducing mothur: Open-source, platform-independent, community-supported software for describing and comparing microbial communities. Appl. Environ. Microbiol..

[20] Kozich J.J., Westcott S.L., Baxter N.T., Highlander S.K., Schloss P.D. (2013). Development of a dual-index sequencing strategy and curation pipeline for analyzing amplicon sequence data on the MiSeq Illumina sequencing platform. Appl. Environ. Microbiol..

[21] Quast C., Pruesse E., Yilmaz P., Gerken J., Schweer T., Yarza P. (2013). The SILVA ribosomal RNA gene database project: Improved data processing and web-based tools. Nucleic Acids Res..

[22] R Core Team (2017). R: A Language and Environment for Statistical Computing.

[23] Kindt R., Coe R. (2005). Tree Diversity Analysis: A Manual and Software for Common Statistical Methods for Ecological and Biodiversity Studies.

[24] Hammer Ø., Harper D.A.T., Ryan P.D. (2001). PAST: Paleontological statistics software package for education and data analysis. Palaeontol. Electron..

[25] Cheng J., Karambelkar B., Xie Y. (2018). Leaflet: Create Interactive Web Maps with the JavaScript ‘Leaflet’ Library.

[26] Gu Z., Gu L., Eils R., Schlesner M., Brors B. (2014). Circlize implements and enhances circular visualization in R. Bioinformatics.

[27] Urbieta M.S. (2013). Diversidad Microbiana en Ambientes Volcánicos. Ph.D. Thesis.

[28] Doménech X. (2000). El Medio Hídrico Terrestre. QUÍMICA de la Hidrósfera.

[29] Legendre P., Legendre L. (1998). Numerical Ecology.

[30] Gammons C.H., Wood S.A., Pedrozo F., Varekamp J.C., Nelson B.J., Shope C.L., Baffico G. (2005). Hydrogeochemistry and rare earth element behavior in a volcanically acidified watershed in Patagonia, Argentina. Chem. Geol..

[31] Fernández-Remolar D.C., Rodriguez N., Gómez F., Amils R. (2003). Geological record of an acidic environment driven by iron hydrochemistry: The Tinto River system. J. Geophys. Res. Planets.

[32] Lavalle L., Chiacchiarini P., Pogliani C., Donati E. (2005). Isolation and characterization of acidophilic bacteria from Patagonia, Argentina. Process Biochem..

[33] Chiacchiarini P., Lavalle L., Giaveno A., Donati E. (2010). First assessment of acidophilic microorganisms from geothermal Copahue–Caviahue system. Hydrometallurgy.

[34] Watanabe T., Kojima H., Fukui M. (2016). Sulfuriferula thiophila sp nov., a chemolithoautotrophic sulfur-oxidizing bacterium, and correction of the name Sulfuriferula plumbophilus Watanabe, Kojima and Fukui 2015 to Sulfuriferula plumbiphila corrig. Int. J. Syst. Evol. Microbiol..

[35] Johnson D.B., Bacelar-Nicolau P., Okibe N., Thomas A., Hallberg K.B. (2009). Ferrimicrobium acidiphilum gen. nov., sp. nov. and Ferrithrix thermotolerans gen. nov., sp. nov.: Heterotrophic, iron-oxidizing, extremely acidophilic actinobacteria. Int. J. Syst. Evol. Microbiol..

[36] Kadnikov V.V., Gruzdev E.V., Ivasenko D.A., Beletsky A.V., Mardanov A.V., Danilova E.V., Karnachuk O.V., Ravin N.V. (2019). Selection of a Microbial Community in the Course of Formation of Acid Mine Drainage. Microbiology.

[37] Sanyika T.W., Stafford W., Cowan D.A. (2012). The soil and plant determinants of community structures of the dominant actinobacteria in Marion Island terrestrial habitats, Sub-Antarctica. Polar Biol..

[38] Marnocha C.L., Dixon J.C. (2014). Bacterially facilitated rock-coating formation as a component of the geochemical budget in cold climates: An example from Kärkevagge, Swedish Lapland. Geomorphology.

[39] Yang Y., Yang L.I., Sun Q.Y. (2014). Archaeal and bacterial communities in acid mine drainage from metal-rich abandoned tailing ponds, Tongling, China. Trans. Nonf. Met. Soc. China.

[40] Giaveno M.A., Urbieta M.S., Ulloa J.R., González-Toril E., Donati E.R. (2013). Physiologic versatility and growth flexibility as the main characteristics of a novel thermoacidophilic Acidianus strain isolated from Copahue geothermal area in Argentina. Microb. Ecol..

[41] Harrison A.P. (1981). Acidiphilium cryptum gen. nov., sp. nov., heterotrophic bacterium from acidic mineral environments. Int. J. Syst. Evol. Microbiol..

[42] Ziegler S., Waidner B., Itoh T., Schumann P., Spring S., Gescher J. (2013). Metallibacterium scheffleri gen. nov., sp. nov., an alkalinizing gammaproteobacterium isolated from an acidic biofilm. Int. J. Syst. Evol. Microbiol..

[43] Lima M.A., Urbieta M.S., Donati E. (2019). Characterization of diverse arsenic-tolerant enrichment cultures from sediments of Copahue geothermal system. J. Basic Microbiol..

[44] Urbieta M.S., González-Toril E., Bazán Á.A., Giaveno M.A., Donati E. (2015). Comparison of the microbial communities of hot springs waters and the microbial biofilms in the acidic geothermal area of Copahue (Neuquén, Argentina). Extremophiles.

[45] Korzhenkov A.A., Toshchakov S.V., Bargiela R., Gibbard H., Ferrer M., Teplyuk A.V., Golyshina O.V. (2019). Archaea dominate the microbial community in an ecosystem with low-to-moderate temperature and extreme acidity. Microbiome.

