# Supplementary material for: A Deeper Look into the Biodiversity of the Extremely Acidic Copahue volcano-Río Agrio System in Neuquén, Argentina
Source: Microorganisms. 2019 Dec 29;8(1):58. doi: 10.3390/microorganisms8010058 (PMC7027000; doi:10.3390/microorganisms8010058)
Supplement: Supplementary file 1 [file microorganisms-08-00058-s001.zip › microorganisms-661678-References.pdf]

microorganisms

MDPI

References

1. Amils, R.; González-Toril, E.; Fernández Remolar, D.; Gómez, F.; Aguilera, A.; Rodríguez, N.; Malki, M.; García-Moyano, A.; Fairén, A.; de la Fuente, V.; et al. Extreme environments as Mars terrestrial analogs: The Rio Tinto case. *Planet. Space Sci.* **2007**, *55*, 370–381, doi:10.1016/j.pss.2006.02.006.
2. González-Toril, E.; Llobet-Brossa, E.; Casamayor, E.O.; Amann, R.; Amils, R. Microbial ecology of an extreme acidic environment, the Tinto River. *Appl. Environ. Microbiol.* **2003**, *69*, 4853–4865, doi:10.1128/AEM.69.8.4853-4865.2003.
3. Garcia-Moyano, A.; Gonzalez-Toril, E.; Aguilera, A.; Amils, R. Comparative microbial ecology study of the sediments and the water column of the Rio Tinto, an extreme acidic environment. *FEMS Microbiol. Ecol.* **2012**, *81*, 303–314, doi:10.1111/j.1574-6941.2012.01346.x.
4. Sánchez-Andrea, I.; Rodríguez, N.; Amils, R.; Sanz, J.L. Microbial diversity in anaerobic sediments at Rio Tinto, a naturally acidic environment with a high heavy metal content. *Appl. Environ. Microbiol*. **2011**, *77*, 6085–6093, doi:10.1128/AEM.00654-11.
5. Bomberg, M.; Mäkinen, J.; Salo, M.; Kinnunen, P. High Diversity in Iron Cycling Microbial Communities in Acidic, Iron-Rich Water of the Pyhäsalmi Mine, Finland. *Geofluids* **2019**, doi:10.1155/2019/7401304.
6. Aguinaga, O.E.; Wakelin, J.F.; White, K.N.; Dean, A.P.; Pittman, J.K. The association of microbial activity with Fe, S and trace element distribution in sediment cores within a natural wetland polluted by acid mine drainage. *Chemosphere* **2019**, *231*, 432–441, doi:10.1016/j.chemosphere.2019.05.157.
7. Gao, P.; Sun, X.; Xiao, E.; Xu, Z.; Li, B.; Sun, W. Characterization of iron-metabolizing communities in soils contaminated by acid mine drainage from an abandoned coal mine in Southwest China. *Environ. Sci. Pollut. Res.* **2019**, *26*, 9585–9598, doi: 10.1007/s11356-019-04336-6.
8. Zhang, X.; Tang, S.; Wang, M.; Sun, W.; Xie, Y.; Peng, H.; Zhong, A.; Liu H.; Zhang, X.; Yu, H.; et al. Acid mine drainage affects the diversity and metal resistance gene profile of sediment bacterial community along a river. *Chemosphere* **2019**, *217*, 790–799, doi:10.1016/j.chemosphere.2018.10.210.
9. Leitholf, A.M.; Fretz, C.E.; Mahanke, R.; Santangelo, Z.; Senko, J.M. An integratemicrobiological and electrochemical approach to determine distributions of Fe metabolism in acid mine drainage-induced "iron mound" sediments. *PLoS ONE* **2019**, *14*, doi:10.1371/journal.pone.0213807.
10. Urbieta, M.S.; González-Toril, E.; Aguilera, A.; Giaveno, M.A.; Donati, E. First prokaryotic biodiversity assessment using molecular techniques of an acidic river in Neuquén, Argentina. *Microb. Ecol.* **2012**, *64*, 91–104, doi:10.1007/s00248-011-9997-2.
11. Jones, D.S.; Schaperdoth, I.; Macalady, J.L. Biogeography of sulfur-oxidizing Acidithiobacillus populations in extremely acidic cave biofilms. *ISME J.* **2016**, *10*, 2879–2882, doi:10.1038/ismej.2016.74.
12. Fontaneto, D.; Hortal, J. Microbial biogeography: Is everything small everywhere. In *Microbial Ecological Theory: Current Perspectives*; Ogilvie, L.A., Hirsch, P.R., Eds.; Caister Academic Press: Norfolk, UK, 2012; pp. 87–98.
13. Holanda, R.; Hedrich, S.; Nancucheo, I.; Oliveira, G.; Grail, B.M.; Johnson, D.B. Isolation and characterisation of mineral-oxidising 'Acidibacillus' spp. from mine sites and geothermal environments in different global locations. *Res. Microbiol.* **2016**, *167*, 613–623, doi:10.1016/j.resmic.2016.04.008.
14. Menzel, P.; Gudbergsdóttir, S.R.; Rike, A.G.; Lin, L.; Zhang, Q.; Contursi, P.; Moracci, M.; Kristjansson, J.K.; Bolduc, B.; Gavrilov, S.; et al. Comparative metagenomics of eight geographically remote terrestrial hot springs. *Microb. Ecol.* **2015**, *70*, 411–424, doi:10.1007/s00248-015-0576-9.
15. Urbieta, M.S.; Willis-Porati, G.; Segretín, A.; González-Toril, E.; Giaveno, M.; Donati, E. Copahue geothermal system: A volcanic environment with rich extreme prokaryotic biodiversity. *Microorganisms* **2015**, *3*, 344–363, doi:10.3390/microorganisms3030344.
16. Varekamp, J.C.; Ouimette, A.; Herman, S.; Flynn, K.S.; Bermúdez, A.H.; Delpino, D.H. Naturally acid waters from Copahue volcano, Argentina. *Appl. Geochem.* **2009**, *24*, 208–220, doi:10.1016/j.apgeochem.2008.11.018.
17. Herrera, A.; Cockell, C.S. Exploring microbial diversity in volcanic environments: A review of methods in DNA extraction. *J. Microb. Methods* **2007**, *70*, 1–12, doi:10.1016/j.mimet.2007.04.005.
18. Ma, Y.J.; Tie, Z.Z.; Zhou, M.; Wang, N.; Cao, X.J.; Xie, Y. Accurate determination of low-level chemical oxygen demand using a multistep chemical oxidation digestion process for treating drinking water samples. *Anal. Methods* **2016**, *8*, 3839–3846, doi:10.1039/C6AY00277C.

19. Schloss, P.D.; Westcott, S.L.; Ryabin, T.; Hall, J.R.; Hartmann, M.; Hollister, E.B.; Sahl, J.W. Introducing mothur: Open-source, platform-independent, community-supported software for describing and comparing microbial communities. *Appl. Environ. Microbiol.* **2009**, *75*, 7537–7541, doi:10.1128/AEM.01541-09.
20. Kozich, J.J.; Westcott, S.L.; Baxter, N.T.; Highlander, S.K.; Schloss, P.D. Development of a dual-index sequencing strategy and curation pipeline for analyzing amplicon sequence data on the MiSeq Illumina sequencing platform. *Appl. Environ. Microbiol.* **2013**, *79*, 5112–5120, doi:10.1128/AEM.01043-13.
21. Quast, C.; Pruesse, E.; Yilmaz, P.; Gerken, J.; Schweer, T.; Yarza, P. The SILVA ribosomal RNA gene database project: Improved data processing and web-based tools. *Nucleic Acids Res.* **2013**, *41*, 590–596, doi:10.1093/nar/gks1219.
22. R Core Team. *R: A Language and Environment for Statistical Computing*; R Foundation for Statistical Computing: Vienna, Austria, 2017. Available online: https://www.R-project.org/ (accessed on)
23. Kindt, R.; Coe, R. *Tree Diversity Analysis: A Manual and Software for Common Statistical Methods for Ecological and Biodiversity Studies*; World Agroforestry Centre (ICRAF): Nairobi, Kenya, 2005; ISBN 92-9059-179-X. Available online: http://www.worldagroforestry.org/output/tree-diversity-analysis (accessed on).
24. Hammer, Ø.; Harper, D.A.T.; Ryan, P.D. PAST: Paleontological statistics software package for education and data analysis. *Palaeontol. Electron.* **2001**, *4*, 9. Available online: http://palaeoelectronica.org/2001_1/past/issue1_01.html (accessed on).
25. Cheng, J.; Karambelkar, B.; Xie, Y. *Leaflet: Create Interactive Web Maps with the JavaScript 'Leaflet' Library*; R package version 2.0.2; 2018. Available online: https://CRAN.R-project.org/package=leaflet (accessed on).
26. Gu, Z.; Gu, L.; Eils, R.; Schlesner, M.; Brors, B. Circlize implements and enhances circular visualization in R. *Bioinformatics* **2014**, *30*, 2811–2812, doi:10.1093/bioinformatics/btu393.
27. Urbieta, M.S. Diversidad Microbiana en Ambientes Volcánicos. Ph.D. Thesis, Universidad Nacional de La Plata, Buenos Aires, Argentina, November 2013, doi:10.35537/10915/30890.
28. Doménech, X. *El Medio Hídrico Terrestre. QUÍMICA de la Hidrósfera*, 3rd ed.; Miraguano: Madrid, Spain, 2000; pp. 17–35.
29. Legendre, P.; Legendre, L. *Numerical Ecology*, 2nd English ed.; Elsevier: Amsterdam, The Netherlands, 1998; Volume 24, p. 853.
30. Gammons, C.H.; Wood, S.A.; Pedrozo, F.; Varekamp, J.C.; Nelson, B.J.; Shope, C.L.; Baffico, G. Hydrogeochemistry and rare earth element behavior in a volcanically acidified watershed in Patagonia, Argentina. *Chem. Geol.* **2005**, *222*, 249–267, doi:10.1016/j.chemgeo.2005.06.002.
31. Fernández-Remolar, D.C.; Rodriguez, N.; Gómez, F.; Amils, R. Geological record of an acidic environment driven by iron hydrochemistry: The Tinto River system. *J. Geophys. Res. Planets* **2003**, *108*, E7, doi:10.1029/2002JE001918.
32. Lavalle, L.; Chiacchiarini, P.; Pogliani, C.; Donati, E. Isolation and characterization of acidophilic bacteria from Patagonia, Argentina. *Process Biochem.* **2005**, *40*, 1095–1099, doi:10.1016/j.procbio.2004.03.008.
33. Chiacchiarini, P.; Lavalle, L.; Giaveno, A.; Donati, E. First assessment of acidophilic microorganisms from geothermal Copahue–Caviahue system. *Hydrometallurgy* **2010**, *104*, 334–341, doi:10.1016/j.hydromet.2010.02.020.
34. Watanabe, T.; Kojima, H.; Fukui, M. Sulfuriferula thiophila sp nov., a chemolithoautotrophic sulfur-oxidizing bacterium, and correction of the name Sulfuriferula plumbophilus Watanabe, Kojima and Fukui 2015 to Sulfuriferula plumbiphila corrig. *Int. J. Syst. Evol. Microbiol.* **2016**, *66*, 2041–2045, doi:10.1099/ijsem.0.000988.
35. Johnson, D.B.; Bacelar-Nicolau, P.; Okibe, N.; Thomas, A.; Hallberg, K.B. Ferrimicrobium acidiphilum gen. nov., sp. nov. and Ferrithrix thermotolerans gen. nov., sp. nov.: Heterotrophic, iron-oxidizing, extremely acidophilic actinobacteria. *Int. J. Syst. Evol. Microbiol.* **2009**, *59*, 1082–1089, doi:10.1099/ijs.0.65409-0.
36. Kadnikov, V.V.; Gruzdev, E.V.; Ivasenko, D.A.; Beletsky, A.V.; Mardanov, A.V.; Danilova, E.V.; Karnachuk, O.V.; Ravin, N.V. Selection of a Microbial Community in the Course of Formation of Acid Mine Drainage. *Microbiology* **2019**, *88*, 292–299, doi:10.1134/S0026261719030056.
37. Sanyika, T.W.; Stafford, W.; Cowan, D.A. The soil and plant determinants of community structures of the dominant actinobacteria in Marion Island terrestrial habitats, Sub-Antarctica. *Polar Biol.* **2012**, *35*, 1129–1141. 10.1007/s00300-012-1160-0.

38. Marnocha, C.L.; Dixon, J.C. Bacterially facilitated rock-coating formation as a component of the geochemical budget in cold climates: An example from Kärkevagge, Swedish Lapland. *Geomorphology* **2014**, *218*, 45–51.
39. Yang, Y.; Yang, L.I.; Sun, Q.Y. Archaeal and bacterial communities in acid mine drainage from metal-rich abandoned tailing ponds, Tongling, China. *Trans. Nonf. Met. Soc. China* **2014**, *24*, 3332–3342, doi:10.1016/S1003-6326(14)63474-9.
40. Giaveno, M.A.; Urbieta, M.S.; Ulloa, J.R.; González-Toril, E.; Donati, E.R. Physiologic versatility and growth flexibility as the main characteristics of a novel thermoacidophilic Acidianus strain isolated from Copahue geothermal area in Argentina. *Microb. Ecol.* **2013**, *65*, 336–346, doi:10.1007/s00248-012-0129-4.
41. Harrison, A.P. Jr. Acidiphilium cryptum gen. nov., sp. nov., heterotrophic bacterium from acidic mineral environments. *Int. J. Syst. Evol. Microbiol.* **1981**, *31*, 327–332, doi:10.1099/00207713-31-3-327.
42. Ziegler, S.; Waidner, B.; Itoh, T.; Schumann, P.; Spring, S.; Gescher, J. Metallibacterium scheffleri gen. nov., sp. nov., an alkalinizing gammaproteobacterium isolated from an acidic biofilm. *Int. J. Syst. Evol. Microbiol.* **2013**, *63*, 1499–1504, doi:10.1099/ijs.0.042986-0.
43. Lima, M.A.; Urbieta, M.S.; Donati, E. Characterization of diverse arsenic-tolerant enrichment cultures from sediments of Copahue geothermal system. *J. Basic Microbiol.* **2019**, *59*, 680–691, doi:10.1002/jobm.201800628.
44. Urbieta, M.S.; González-Toril, E.; Bazán, Á.A.; Giaveno, M.A.; Donati, E. Comparison of the microbial communities of hot springs waters and the microbial biofilms in the acidic geothermal area of Copahue (Neuquén, Argentina). *Extremophiles* **2015**, *19*, 437–450, doi:10.1007/s00792-015-0729-2.
45. Korzhenkov, A.A.; Toshchakov, S.V.; Bargiela, R.; Gibbard, H.; Ferrer, M.; Teplyuk, A.V.; Golyshina, O.V. Archaea dominate the microbial community in an ecosystem with low-to-moderate temperature and extreme acidity. *Microbiome* **2019**, *7*, 11–25, doi:10.1186/s40168-019-0623-8.
46. Battaglia-Brunet, F.; Joulian, C.; Garrido, F.; Dictor, M.C.; Morin, D.; Coupland, K.; Barrie Johnson, D.; Hallberg, K.B.; Baranger, P. Oxidation of arsenite by Thiomonas strains and characterization of Thiomonas arsenivorans sp. nov. *Antonie Leeuwenhoek* **2006**, *89*, 99–108, doi:10.1007/s10482-005-9013-2.
47. Fujimura, R.; Sato, Y.; Nishizawa, T.; Nanba, K.; Oshima, K.; Hattori, M.; Ohta, H. Analysis of early bacterial communities on volcanic deposits on the island of Miyake (Miyake-jima), Japan: A 6-year study at a fixed site. *Microbes Environ.* **2012**, *1*, 19–29, doi:10.1264/jsme2.ME11207.
48. Vorob'ev, A.V.; de Boer, W.; Folman, L.B.; Bodelier, P.L.; Doronina, N.V.; Suzina, N.E.; Trotsenko, Y.A.; Dedysh, S.N. Methylovirgula ligni gen. nov., sp. nov., an obligately acidophilic, facultatively methylotrophic bacterium with a highly divergent mxaF gene. *Int. J. Syst. Evol. Microbiol.* **2009**, *59*, 2538–2545, doi:10.1099/ijs.0.010074-0.
49. Acosta, M.; Galleguillos, P.; Ghorbani, Y.; Tapia, P.; Contador, Y.; Velásquez, A.; Espoz, C.; Pinilla, C.; Demergasso, C. Variation in microbial community from predominantly mesophilic to thermotolerant and moderately thermophilic species in an industrial copper heap bioleaching operation. *Hydrometallurgy* **2014**, *150*, 281–289, doi:10.1016/j.hydromet.2014.09.010.
50. He, Z.; Xiao, S.; Xie, X.; Hu, Y. Microbial diversity in acid mineral bioleaching systems of dongxiang copper mine and Yinshan lead-zinc mine. *Extremophiles* **2008**, *12*, 225–234, doi:10.1007/s00792-007-0130-x.
51. Mendez, M.O.; Neilson, J.W. Characterization of a bacterial community in an abandoned semiarid lead-zinc mine tailing site. *Appl. Environ. Microbiol.* **2008**, *74*, 3899–3907, doi:10.1128/AEM.02883-07.
52. Henneberger, R.M. The Microbial Diversity and Ecology of Selected Andesitic Hydrothermal Environments. Ph.D. Dissertation, Macquarie University, Sidney, Australia, 2008.
53. Coram, N.J.; Rawlings, D.E. Molecular relationship between two groups of the genus Leptospirillum and the finding that Leptospirillum ferriphilum sp. nov. dominates South African commercial biooxidation tanks that operate at 40 degrees C. *Appl. Environ. Microbiol.* **2002**, *68*, 838–845, doi:10.1128/AEM.68.2.838-845.2002.
54. Korehi, H.; Blothe, M.; Schippers, A. Microbial diversity at the moderate acidic stage in three different sulfidic mine tailings dumps generating acid mine drainage. *Res. Microbiol.* **2014**, *165*, 713–718, doi:10.1016/j.resmic.2014.08.007.
55. Gavrilov, S.N.; Korzhenkov, A.A.; Kublanov, I.V.; Bargiela, R.; Zamana, L.; Popova, A.; Toshchakov, S.V.; Golyshin, P.N.; Golyshina, O.V. Microbial communities of polymetallic deposits' acidic ecosystems of continental climatic zone with high temperature contrasts. *Front. Microbiol.* **2019**, *10*, 1573, doi:10.3389/fmicb.2019.01573.

56. Urbieta, M.S.; Toril, E.G.; Giaveno, M.A.; Bazan, A.A.; Donati, E.R. Archaeal and bacterial diversity in five different hydrothermal ponds in the Copahue region in Argentina. *Syst. Appl. Microbiol.* **2014**, *37*, 429–441.
57. Harris, J.K.; Kelley, S.T.; Pace, N.R. New perspective on uncultured bacterial phylogenetic division OP11. *Appl. Environ. Microbiol*. **2004**, *70*, 845–849, doi:10.1128/AEM.70.2.845-849.2004.
58. Liljeqvist, M.; Sundkvist, J.E.; Saleh, A.; Dopson, M. Low temperature removal of inorganic sulfur compounds from mining process waters. *Biotechnol. Bioeng*. **2011**, *108*, 1251–1259, doi:10.1002/bit.23057.
59. Lu, S.; Chourey, K.; Reiche, M.; Nietzsche, S.; Shah, M.B.; Neu, T.R.; Hettich, R.L.; Kusel, K. Insights into the structure and metabolic function of microbes that shape pelagic iron-rich aggregates ('iron snow'). *Appl. Environ. Microbiol*. **2013**, *79*, 4272–4281, doi:10.1128/AEM.00467-13.
