# Supplementary figures and images for: A Deeper Look into the Biodiversity of the Extremely Acidic Copahue volcano-Río Agrio System in Neuquén, Argentina
Source: Microorganisms. 2019 Dec 29;8(1):58. doi: 10.3390/microorganisms8010058 (PMC7027000; doi:10.3390/microorganisms8010058)

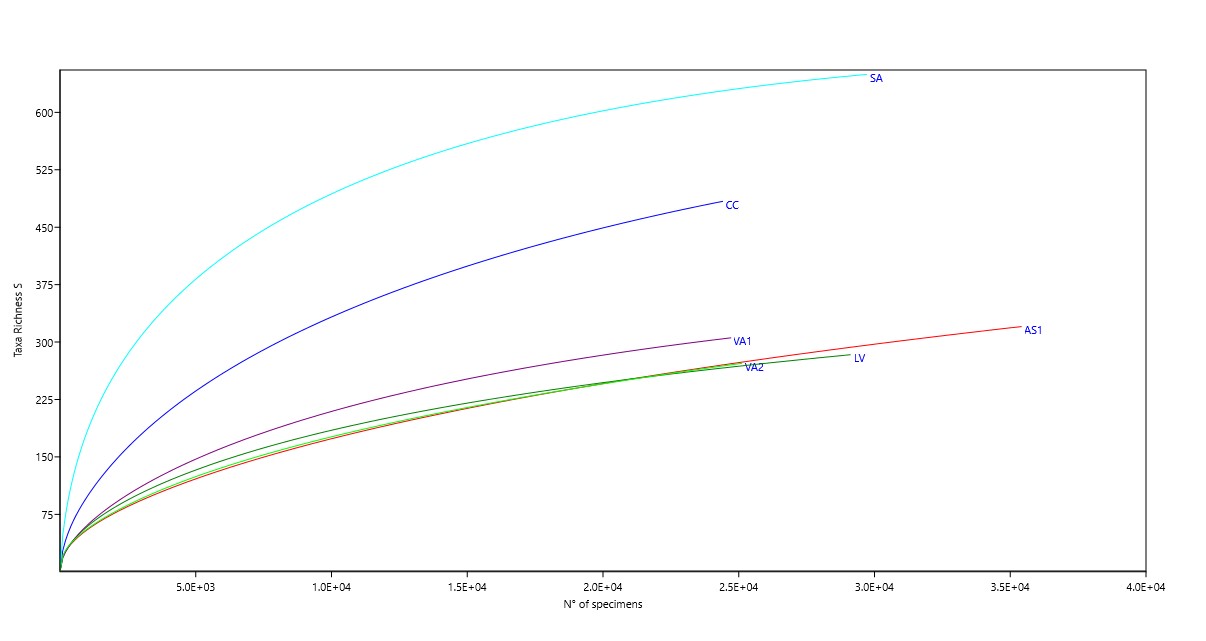

Supplement: Supplementary file 1 [file microorganisms-08-00058-s001.zip › microorganisms-661678- Figure S1.jpg]
